# Association between normal saline infusion volume in the emergency department and acute kidney injury in heat stroke patients: a multicenter retrospective study

**DOI:** 10.1080/0886022X.2023.2294151

**Published:** 2024-01-04

**Authors:** Lan Chen, Junlu Zhao, Liyun Lu, Zhumei Gong, Shuying Xu, Xiaoling Yang, Yuping Zhang, Xiuqin Feng

**Affiliations:** a Nursing Department, The Second Affiliated Hospital of Zhejiang University School of Medicine, Hangzhou, China; b Emergency Department, Affiliated Jinhua Hospital, Zhejiang University School of Medicine, Jinhua Municipal Central Hospital, Jinhua, China; c Emergency Department, Jinhua People’s Hospital, Jinhua, China; d Emergency Department, Yiwu Central Hospital, Yiwu, China; e Emergency Department, Dongyang People’s Hospital, Dongyang, China; f Emergency Department, Lanxi People’s Hospital, Lanxi, China

**Keywords:** Normal saline, acute kidney injury, serum chloride, heat stroke, emergency department

## Abstract

**Background:**

Previous studies have shown that intravenous normal saline (NS) may be associated with the incidence of acute kidney injury (AKI). This study aimed to evaluate the association between the volume of NS infusion and AKI in heat stroke (HS) patients.

**Methods:**

This multicenter retrospective cohort study included 138 patients with HS. The primary outcome was the incidence of AKI. Secondary outcomes included the need for continuous renal replacement therapy (CRRT), admission to the intensive care unit (ICU), length of stay in the ICU and hospital, and in-hospital mortality. Multivariate regression models, random forest imputation, and genetic and propensity score matching were used to explore the relationship between NS infusion and outcomes.

**Results:**

The mean volume of NS infusion in the emergency department (ED) was 3.02 ± 1.45 L. During hospitalization, 33 patients (23.91%) suffered from AKI. In the multivariate model, as a continuous variable (per 1 L), the volume of NS infusion was associated with the incidence of AKI (OR, 2.51; 95% CI, 1.43–4.40; *p* = .001), admission to the ICU (OR, 3.46; 95% CI 1.58–7.54; *p* = .002), and length of stay in the ICU (*β*, 1.00 days; 95% CI, 0.44–1.56; *p* < .001) and hospital (*β*, 1.41 days; 95% CI, 0.37–2.45; *p* = .008). These relationships also existed in the forest imputation cohort and matching cohort. There were no differences in the use of CRRT or in-hospital mortality.

**Conclusions:**

The volume of NS infusion was associated with a significant increase in the incidence of AKI, admission to the ICU, and length of stay in the ICU and hospital among patients with HS.

## Introduction

Heat stroke (HS) is a life-threatening illness caused by a rapid increase in one’s core temperature in excess of 40 °C from exposure to a hot and humid environment [1]. HS manifests as a systemic illness that includes encephalopathy, coagulopathy, respiratory failure, hypotension, liver, kidney and muscle injury, vomiting and diarrhea [2]. Acute kidney injury (AKI) is one of the most common complications of HS, with an incidence of up to 90.9% [3]. AKI following HS is a major factor associated with the need for renal replacement therapy, longer hospital stay, and hospital mortality [4–6].

Immediate cooling and support of organ system function are the two main therapeutic objectives for patients with HS [7]. Crystalloid solution administration is widely considered an essential part of the management of HS. Administering an intravenous cold infusion quickly is an effective cooling method in hospitals [8]. Fluid resuscitation with crystalloid solution is an important intervention to restore organ perfusion, improve tissue oxygenation, promote renal blood flow, and prevent myoglobin-induced renal injury [7]. Normal saline (NS), a common type of crystalloid solution, is widely used in patients with HS, especially in the emergency department (ED) where the patient is in the early and key stage of the disease.

However, the chloride concentration of NS (154 mmol/L) is higher than that of human plasma (94–111 mmol/L) [9]. Chloride-rich fluid administration may induce or exacerbate hyperchloremia and metabolic acidosis [10–12] and may produce renal vasoconstriction and a reduction in the glomerular filtration rate [13, 14]. Recent large-scale studies have brought forward compelling evidence on this matter. Some indicate that compared to balanced crystalloids, NS might be associated with a higher incidence of adverse kidney events in both critical and non-critical adult cases [9, 15]. There have been suggestions that moderating intravenous chloride intake might lead to a reduced AKI incidence and lowered necessity for continuous renal replacement therapy (CRRT) in critically ill patients [9, 16]. Yet, the debate continues. Other extensive trials have posited that balanced solutions might not significantly outperform NS in reducing mortality or AKI in critically ill patients [17]. Likewise, systematic reviews have also hinted at negligible differences in outcomes between NS and balanced solutions for septic adults [18].

Given this backdrop, the association between NS administration and AKI remains hotly debated. Complicating matters further is the fact that many large-scale studies have encompassed a diverse patient pool [9, 15–17], leaving gaps in our understanding regarding NS’s effects in specific conditions like HS. Recognizing the unique pathophysiological nuances of HS and the potential implications of crystalloid solution choice, our research endeavors to shed light on the relationship between NS infusion volume and HS outcomes. Our retrospective multicenter cohort study, spanning 2021 to 2022, delves into this relationship, aiming to offer valuable insights and guiding future HS management strategies.

## Methods

This study was approved by the Ethics Committee of the Second Affiliated Hospital of Zhejiang University School of Medicine (approval number: 2022-0913), and it conforms to the provisions of the Declaration of Helsinki. The requirement for informed consent was waived due to the retrospective nature of the study. The STROBE guidelines for cohort studies were applied.

### Study design, setting, and participants

This retrospective cohort study was conducted in six tertiary care hospitals in China between 2021 and 2022. Consecutive adult patients with HS who were admitted to EDs were included in our study. HS was defined by the criteria according to the expert consensus on the diagnosis and treatment of HS in China [19]. Patients were excluded from the study for several reasons: (1) if they were transferred from another hospital or if the volume of NS infused prior to admission was undocumented; (2) if they had existing end-stage renal disease on chronic dialysis; and (3) if initial laboratory results at ED admission indicated an imminent risk for AKI. In line with the Clinical Practice Guidelines [20] and taking into account the typical hypovolemia in HS, the exclusion criteria were as follows: a serum creatinine level exceeding 140 mmol/L; (4) concurrent critical conditions such as severe trauma or substantial intracranial hemorrhage; and (5) self-discharge within the first 24 h of admission due to the severity of their condition, leaving outcome data incomplete.

### Data collection and management

We collected information on patients’ baseline characteristics, underlying comorbidities, state of consciousness and vital signs obtained at ED admission, and length of stay in the ED. The National Early Warning Score (NEWS), a critical severity scoring system commonly used in EDs, was calculated based on vital signs and GCS obtained at ED admission, as well as supplemental oxygen [21]. Data from laboratory tests performed at ED admission were extracted. We retrieved the first serum chloride result after admission to the intensive care unit (ICU) or general ward. If the patient was discharged from the ED, then the last test results before discharge were retrieved. Specific treatments, such as the use of vasoactive drugs, and CRRT, regardless of whether it is due to AKI or non-AKI reasons, were also extracted from the electronic patient data. The volume of NS infusion in the ED was rechecked between the doctor’s orders and the drug administration list. That during prehospital care was calculated according to the nursing record sheet.

### Outcomes

The primary outcome of this study was the incidence of AKI. AKI was defined as an increase in serum creatinine by 50% within seven days, an increase in serum creatinine by 0.3 mg/dL (26.5 μmol/L) within two days, or oliguria [22]. The secondary outcomes included admission to the ICU, the need for CRRT, length of stay in the ICU and hospital, and in-hospital mortality.

### Statistical analysis

All statistical analyses were performed using Empower (R) (www.empowerstats.com, X&Y Solutions, Inc., Boston, MA). A two-tailed *p* Value <.05 was considered statistically significant.

The patients’ baseline characteristics were summarized using descriptive statistics. Categorical variables are reported as absolute numbers with percentages and were analyzed by Chi-square or Fisher’s exact test, as appropriate. Normally and nonnormally distributed continuous variables were compared using Student’s *t*-test and the Mann–Whitney’s *U*-test, respectively.

The incidence of AKI was analyzed using logistic regression, and the results are reported as odds ratios (ORs) with 95% confidence intervals (CIs). Multivariable sensitivity analysis was performed for all outcomes. After screening for collinearity between covariates according to the variance inflation factor, covariates were included as potential confounders in the adjusted models if the changes in the estimates of the NS infusion volume for the outcomes were more than 10% or were significantly associated with the outcomes. The associations of each confounder with the outcomes of interest and changes in the effect estimates are shown in Supplementary Tables S1–S3. The serum creatinine level obtained at ED admission was also included as a covariate for all outcomes since late renal function was greatly dependent on the starting level.

There was not a great deal of missing data in our study. The largest amount of missing data in potential confounders was lactic acid level (7, 5.07%). We used random forest imputation, based on five replications, to account for missing data on covariates. We repeated logistic regression analysis with the complete data cohort to assess the association between the NS infusion volume and AKI for sensitivity analyses. Supplementary Tables S4 and S5 give additional details of the statistical analyses.

Furthermore, we divided the participants into two groups: a group with a volume of NS >2.5 L and a group with a volume ≦2.5 L. As the baseline data between the groups with different volumes of NS were not balanced, the patients with a greater NS infusion volume showed more severe conditions, and genetic matching incorporating the estimated propensity score was performed to adjust for confounders and balance the observed covariates between these two groups. Specifically, we used institution and baseline characteristics, HS type, NEWS at admission, baseline laboratory results, length of stay in the ED, and use of vasoactive drugs as covariates, calculated the propensity score for each patient, and performed 1:1 matching using a genetic matching algorithm. Genetic matching, a method for multivariate matching, leverages an affinely invariant approach [23]. The method utilizes an evolutionary algorithm, meticulously assigning weights to a range of baseline covariates. This strategic weighting is designed to ensure the optimal balance of these covariates, thereby achieving a state of equilibrium between the matched treatment and control groups in observational studies. While the methodology is nonparametric and independent of the propensity score’s estimation or knowledge, its efficiency is significantly enhanced when the propensity score is either known or estimated [23]. Multivariate regression model was conducted to assess differences in outcomes between the two groups after matching. The propensity score was included as a covariate in the adjusted models.

To further explore the possible mechanism between the NS infusion volume and outcomes, we analyzed the correlation between the changes in chloride and the volume of saline infusion to assess the possible immediate effect of saline infusion. Then, we performed analyses of the association between serum chloride, including the baseline level and the level tested after NS infusion, and the incidence of AKI. Moreover, we used a smoothing spline curve to characterize the risk of AKI associated with chloride after saline infusion.

## Results

**Figure 1.**
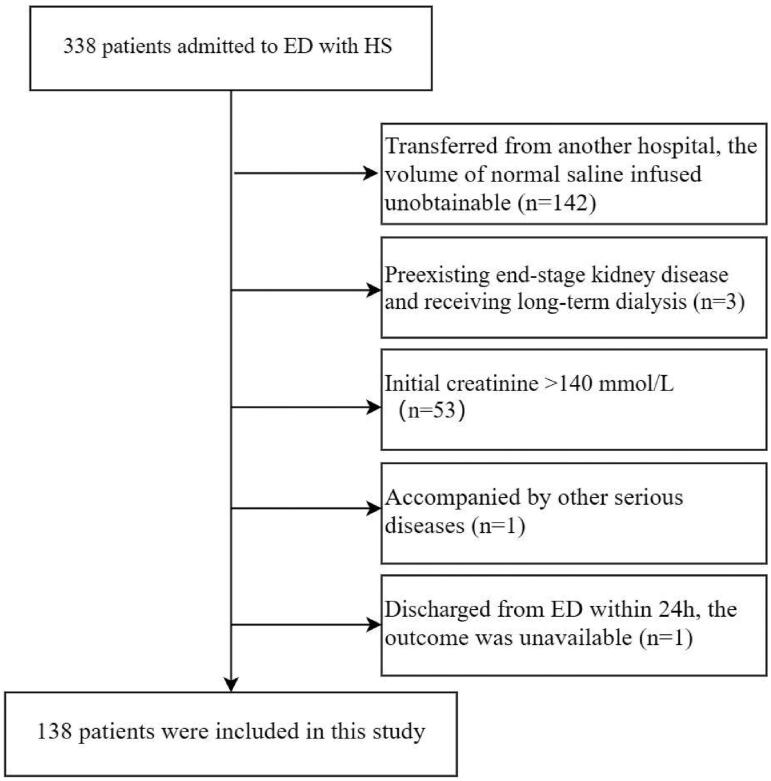
Flowchart.

In the final analysis, 138 patients were included (Figure 1). Baseline demographics, vital signs obtained at ED admission, laboratory tests, use of CRRT, use of vasoactive drugs, and complications are outlined in Table 1.

**Table 1.** Characteristics of the patients.

Variables	All (*n* = 138)	Acute kidney injury		*p*
		No (*n* = 105)	Yes (*n* = 33)	
Baseline characteristics				
Age (years)	65.26 ± 14.84	66.46 ± 14.57	61.45 ± 15.26	.068
Female, *n* (%)	55 (39.86)	42 (40.00)	13 (39.39)	.951
Comorbidities, *n* (%)				
Diabetes	9 (6.52)	5 (4.76)	4 (12.12)	.135
Chronic cardiovascular disease	40 (28.99)	31 (29.52)	9 (27.27)	.977
Mental disorder	14 (10.14)	10 (9.52)	4 (12.12)	.666
Dementia	5 (3.62)	4 (3.81)	1 (3.03)	.834
Institution, *n* (%)				.030
1	47 (34.0)	40 (38.10)	7 (21.21)	
2	19 (13.77)	13 (12.38)	6 (18.18)	
3	22 (15.94)	17 (16.19)	5 (15.15)	
4	15 (10.87)	14 (13.33)	1 (3.03)	
5	22 (15.94)	15 (14.29)	7 (21.21)	
6	13 (9.42)	6 (5.71)	7 (21.21)	
Type of heat stroke, *n* (%)				.555
Classic	52 (37.68)	41 (39.05)	11 (33.33)	
Exertional	86 (62.32)	64 (60.95)	22 (66.67)	
Chief complaints, *n* (%)				
Coma	113 (81.88)	82 (78.10)	31 (93.94)	.039
Epileptic seizure	19 (13.77)	12 (11.43)	7 (21.21)	.155
Onset time (h)	1.00 (1.00–3.00)	1.50 (1.00–3.00)	1.00 (1.00–3.00)	.470
Monitoring parameters at admission				
Temperature (°C)	40.80 ± 0.81	40.77 ± 0.77	40.89 ± 0.95	.452
Heart rate (beat/min)	128.57 ± 24.08	124.41 ± 23.64	141.82 ± 20.68	<.001
Systolic pressure (mmHg)	126.62 ± 31.54	130.30 ± 31.42	114.94 ± 29.44	.014
Glasgow Coma Scale	8.80 ± 4.19	9.50 ± 4.29	6.58 ± 2.94	<.001
NEWS	12.51 ± 3.59	11.93 ± 3.69	14.36 ± 2.55	<.001
Body temperature after cooling (°C)				
Temperature (0.5 h)	39.50 ± 0.99	39.32 ± 0.93	40.01 ± 0.90	<.001
Temperature (2 h)	37.92 ± 1.00	37.73 ± 0.91	38.49 ± 1.05	<.001
Blood test at admission				
C-reactive protein (mg/L)	0.62 (0.30–4.40)	0.70 (0.30–5.80)	0.30 (0.30–2.0)	.458
Potassium (mmol/L)	3.64 ± 0.63	3.58 ± 0.60	3.83 ± 0.70	.053
Sodium (mmol/L)	130.46 ± 13.07	130.86 ± 7.67	129.21 ± 23.19	.529
Chloride (mmol/L)	97.54 ± 9.06	97.11 ± 8.93	98.89 ± 9.49	.329
Blood glucose (mmol/L)	11.35 ± 4.87	10.90 ± 4.80	12.70 ± 4.90	.065
Alanine aminotransferase (IU/L)	23 (16.25–35.00)	22.50 (16.25–35)	23 (17.00–32.58)	.620
Creatinine (mmol/L)	98.11 ± 23.35	93.45 ± 23.26	112.92 ± 16.72	<.001
Blood urea nitrogen (mmol/L)	6.86 ± 2.73	6.88 ± 2.82	6.80 ± 2.45	.882
Creatine kinase (U/L)	265.50 (141.80–703.75)	256.00 (133.20–546.55)	395.00(159.50–930.00)	.656
Troponin I (ng/mL)	0.03 (0.01–0.07)	0.02 (0.01–0.06)	0.04 (0.02–0.15)	.061
Prothrombin time (s)	13.25 ± 2.01	13.29 ± 1.90	13.12 ± 2.37	.681
Lactic acid (mmol/L)	3.42 ± 2.00	3.18 ± 2.08	4.15 ± 1.53	.016
White blood cell count (1 × 10^9^/L)	10.24 ± 4.71	10.25 ± 4.70	10.23 ± 4.82	.983
Platelet (1 × 10^9^/L)	197.01 ± 71.15	194.94 ± 69.07	203.55 ± 78.12	.547
Serum chlorine after normal saline infusion (mmol/L)	107.67 ± 6.96	106.30 ± 6.53	110.83 ± 6.99	.008
Volume of normal saline infusion in ED (L)	3.02 ± 1.45	2.82 ± 1.34	3.66 ± 1.61	.006
Total infusion volume (L)	3.69 ± 1.99	3.48 ± 1.82	4.36 ± 2.19	.043
Use of vasoactive drugs, *n* (%)	28 (20.29)	13 (12.38)	15 (45.45)	<.001
Length of stay in ED (h)	10.00 (4.50–19.88)	10.00 (4.50–22.00)	8.00 (4.00–16.50	.160
Outcomes				
RRT, *n* (%)	7 (5.07)	2 (1.90)	5 (15.15)	.002
Admitted to ICU, *n* (%)	52 (37.68)	25 (23.81)	27 (81.82)	<.001
Length of stay in hospital (d)	5.00 (2.00–8.00)	5.00 (0.00–7.00)	7.00 (5.00–12.00)	.005
Length of stay in ICU (d)	0.00 (0.00–4.00)	0.00 (0.00–0.00)	5.00 (3.00–8.00)	<.001
Coagulation impairment, *n* (%)	20 (14.49)	6 (5.71)	14 (42.42)	<.001
Rhabdomyolysis, *n* (%)	8 (5.80)	6 (5.71)	2 (6.06)	.941
Mortality, *n* (%)	10 (7.25)	5 (4.76)	5 (15.15)	.045
Central nervous system damage at discharge, *n* (%)	16 (11.59)	9 (8.57)	7 (21.21)	.048

NEWS: National Early Warning Score; ED: emergency department; ICU: intensive care unit; RRT: renal replacement therapy.

The mean volume of NS infusion in the ED was 3.02 ± 1.45 L. During hospitalization, 33 patients (23.9%) suffered from AKI, 7 patients (5.07%) needed CRRT, 52 patients (37.68%) were admitted to the ICU, and 10 patients (7.25%) died from all causes. Compared with non-AKI patients, AKI patients had a faster heart rate, lower systolic pressure, lower Glasgow Coma Scale (GCS) score, and higher NEWS. After cooling, AKI patients had higher body temperature at 0.5 h and 2 h. They also had higher levels of creatinine and lactic acid. Patients with AKI were more likely to use vasoactive drugs, receive RRT, be admitted to the ICU, suffer from central nervous system damage at discharge, and have a longer length of stay in the hospital and ICU.

The association between the NS infusion volume and the incidence of AKI was revealed by univariate and multivariate logistic regression (Table 2). As a continuous variable, each liter increase in NS administered was associated with a 46% increase in the odds of AKI in univariate analysis (OR, 1.46; 95% CI, 1.12–1.92; *p* = .005). In the multivariate model, this relationship between the NS infusion volume and the incidence of AKI appeared more significant (OR, 2.51; 95% CI, 1.43–4.40; *p* = .001). The finding was consistent in the random forest imputation cohort (Supplementary Table S5). In addition, all participants were stratified into two groups according to the volume of NS infusion (>2.5 L and ≦2.5 L). The OR of the incidence of AKI was significantly higher in the high volume group than in the low volume group in univariate and adjusted model logistic regression analyses (*p* < .05).

**Table 2.** Logistic regression of normal saline infusion volume in emergency department for acute kidney injury in patients with heat stroke.

Volume of normal saline infusion	Acute kidney injury					
	Univariate		Adjust model I		Adjust model II	
	OR (95% CI)	*p*	OR (95% CI)	*p*	OR (95% CI)	*p*
Per 1 L increase	1.46 (1.12, 1.92)	.005	1.39 (1.03, 1.89)	.033	2.51 (1.43, 4.40)	.001
≦2.5 L	Reference		Reference		Reference	
>2.5 L	4.25 (1.62, 11.14)	.003	5.29 (1.66, 16.85)	.004	59.90 (7.55, 475.08)	<.001

NEWS: National Early Warning Score; OR: odds ratio; CI: confidence interval.

Adjust model I adjusted for sex, age, type of heat stroke, and creatinine. Adjust model II adjusted for institution, NEWS, creatinine, troponin I, lactic acid, chlorine, coronary heart disease, use of vasoactive drugs, and length of stay in emergency department.

The relationships between the NS volume and use of CRRT, admission to the ICU, length of stay in the ICU and hospital, and hospital mortality were revealed by univariate and multivariate regression (Table 3). With increasing volume of NS, the incidence of ICU admission increased, with an OR of 3.46 (95% CI, 1.58–7.54; *p* = .02), while the length of stay in the ICU and hospital also increased, with *β* values of 1.00 days (95% CI, 0.44–1.56; *p* < .001) and 1.41 days (95% CI, 0.37–2.45; *p* = .008), respectively. There were no differences in the use of CRRT or hospital mortality.

**Table 3.** Multivariate regression of normal saline infusion in emergency department for the second outcomes in patients with heat stroke.

Volume of normal saline infusion (L)	OR/*β* (95% CI) *p*		
	Univariate	Adjust model I	Adjust model II
RRT	0.93 (0.54, 1.61) .793	0.70 (0.37, 1.34) .280	0.40 (0.13, 1.22) .107^a^
Admitted to ICU	1.50 (1.16, 1.94) .002	1.43 (1.08, 1.91) .013	3.46 (1.58, 7.54) .002^b^
Length of stay in ICU	1.18 (0.70, 1.67) <.001	1.22 (0.70, 1.74) <.001	1.00 (0.44, 1.56) <.001^c^
Length of stay in hospital	2.52 (1.74, 3.29) <.001	2.64 (1.85, 3.43) <.001	1.41 (0.37, 2.45) .008^d^
Mortality	1.54 (1.04, 2.27) .030	2.08 (1.23, 3.53) .006	1.96 (0.72, 5.35) .189^e^

NEWS: National Early Warning Score; RRT: renal replacement therapy; ICU: intensive care unit; OR: odds ratio; CI: confidence interval.

Adjust model I adjusted for sex, age, type of heat stroke, creatinine.

Adjust model II adjusted for:

^a^ Institution, NEWS, creatinine, lactic acid, and use of vasoactive drugs.

^b^ Institution, sex, age, NEWS, chlorine, creatinine, lactic acid, use of vasoactive drugs, and length of stay in emergency department.

^c^ Institution, type of heat stroke, age, NEWS, creatinine, lactic acid, use of vasoactive drugs, and length of stay in emergency department.

^d^ Institution, type of heat stroke, age, NEWS, creatinine, lactic acid, use of vasoactive drugs, and length of stay in emergency department.

^e^ Institution, type of heat stroke, sex, age, NEWS, chlorine, creatinine, troponin I, use of vasoactive drugs, and length of stay in emergency department.

Genetic and propensity score matching were used to determine the optimal balance between the NS >2.5 L and ≦2.5 L groups. In total, 108 pairs were matched. Table S6 shows the characteristics of the initial and matched cohorts, and Figure S1 is the histogram of PS for two groups. Apart from institution, all other confounders were similar in the matched cohort. Multivariate regression analysis was performed to assess the association between the volume of NS and outcomes. After adjusting for propensity scores, patients who received more than 2.5 L of NS exhibited a higher incidence of AKI (OR, 7.39; 95% CI, 3.01–18.11; *p* < .001), ICU admission (OR, 3.37; 95% CI, 1.91–5.97; *p* < .001), along with longer ICU (*β*, 3.06 days; 95% CI, 2.06–4.05; *p* < .001) and hospital stays (*β*, 3.70 days; 95% CI, 2.34–5.07; *p* < .001), compared to those who received less than 2.5 L of NS (Table 4).

**Table 4.** Multivariate regression of volume of normal saline infusion with outcomes in the genetic matching cohort^a^.

Volume of normal saline infusion	Univariate		Adjust model	
	OR/*β* (95% CI)	*p* Value	OR/*β* (95% CI)	*p* Value
≦2.5 L	Reference		Reference	
>2.5 L				
AKI	6.35 (2.66, 15.13)	<.001	7.39 (3.01, 18.11)	<.001
Admitted to ICU	3.17 (1.81, 5.54)	<.001	3.37 (1.91, 5.97)	<.001
Length of stay in ICU	2.81 (1.78, 3.85)	<.001	3.06 (2.06, 4.05)	<.001
Length of stay in hospital	3.57 (2.21, 4.94)	<.001	3.70 (2.34, 5.07)	<.001

OR: odds ratio; CI: confidence interval: ICU: intensive care unit.

Adjust model adjusted for: propensity-score.

^a^ The genetic matching cohort included 108 patients with volume of normal saline infusion ≦2.5 L, and 108 volume of normal saline infusion >2.5 L.

**Figure 2.**
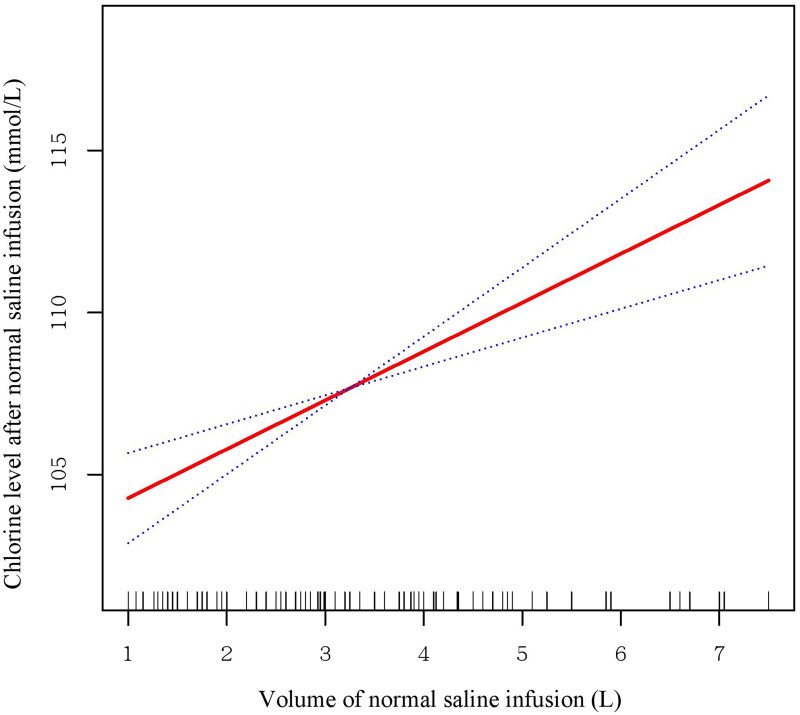
Smoothing spline curve of the relationship between the NS infusion volume and serum chloride levels after saline infusion. Increasing NS infusion volume was significantly associated with increasing serum chloride levels after NS infusion.

**Figure 3.**
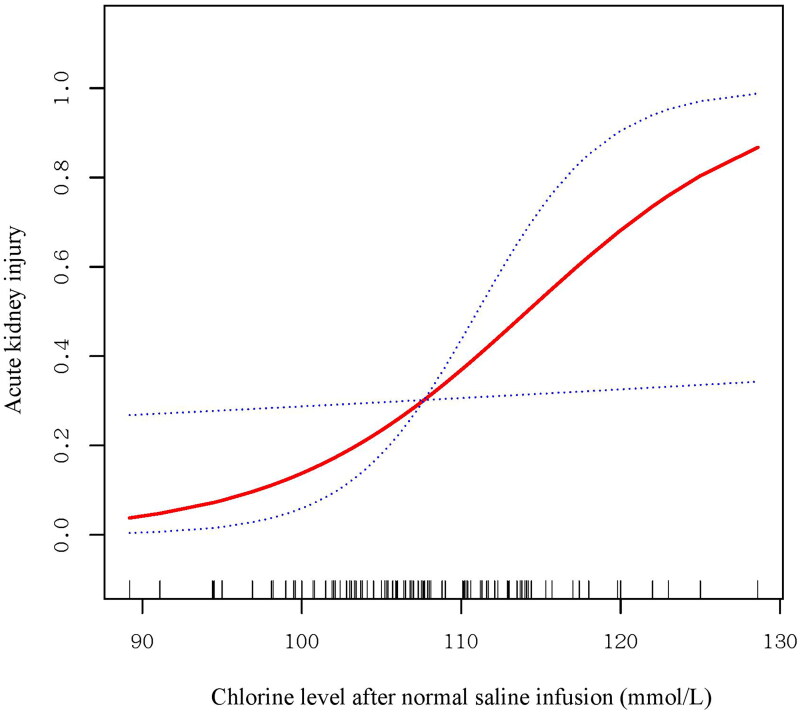
Smoothing spline curve of the association between serum chloride levels after saline infusion and AKI. The relationship between chloride and AKI showed a nearly S-shaped curve. The incidence of AKI increased sharply with increasing chloride when the chloride level was over 100 mmol/L.

Moreover, we found that the chloride level significantly increased with increasing NS volume (Figure 2). The chloride level after NS infusion was significantly associated with the incidence of AKI (OR 1.14; 95% CI, 1.01–1.28; *p* = .040) (Table S7). As shown in Figure 3, the relationship between chloride and AKI showed a nearly S-shaped curve. The incidence of AKI increased sharply with increasing chloride level when the chloride level was over 100 mmol/L. However, a relationship could not be found between baseline chloride level and the incidence of AKI in either the univariate or adjusted model.

## Discussion

We performed a retrospective multicenter cohort study to explore the association between the volume of NS infusion in the ED and the incidence of AKI among patients with HS. We found that NS volume was associated with a significant increase in the incidence of AKI. In addition, we found that the NS infusion volume was associated with admission to the ICU and the length of stay in the ICU and hospital. Moreover, the chloride level after NS infusion was significantly associated with the incidence of AKI. These findings remained significant after adjusting for baseline variables.

HS is often accompanied by serious clinical syndromes and multiorgan dysfunction and has a high fatality rate [19, 1]. Renal dysfunction is common in both exertional HS (EHS) and classic HS (CHS) [3, 24–26]. In our study cohort, after excluding patients with significant abnormalities in the initial creatinine level, 33 (23.91%) patients suffered from AKI, 11 (21.15%) from CHS, and 22 (25.58%) from EHS. In HS management, blood purification plays a crucial role. It serves not just for AKI management but also as an efficacious intravascular cooling method. Additionally, it aids in the elimination of proinflammatory cytokines associated with HS and can benefit patients with severe HS-induced liver damage [1, ]. Consequently, two (1.9%) non-AKI patients underwent RRT in our cohort.

Renal damage in HS patients is multifactorial, involving direct thermal injury, hypovolemia, renal hypoperfusion, rhabdomyolysis, and DIC [8]. In our study’s cohort, the body temperatures at 0.5 h and 2 h for the AKI group were significantly elevated compared to the non-AKI group, highlighting the impact of hyperthermia on organ damage. Patients with AKI displayed notably more rapid heart rate, reduced systolic blood pressure, and a greater propensity for the administration of vasoactive agents, suggesting inadequate blood volume and renal hypoperfusion. Additionally, the likelihood of coagulopathy was markedly increased in AKI patients. Interestingly, rhabdomyolysis rates did not differ significantly between patients with and without AKI, possibly due to our study’s exclusion criteria. Among the 53 excluded patients, eight (15.09%) developed rhabdomyolysis, which was a higher incidence rate than in the cohort of this study.

Rapid administration of cold NS is a highly effective *in vivo* cooling technique for HS patients [2, ], notably decreasing body temperature and hospital stay durations [27]. This method, particularly recommended for dehydrated EHS patients, is endorsed by experts in China for its safety and efficiency [3]. Our study found that 97.1% of HS patients received this treatment. Moreover, HS often leads to hypovolemic shock due to factors like excessive sweating and diarrhea [2, 28]. Thus, intravenous isotonic crystalloids, especially sodium-containing solutions like NS, are key for effective volume resuscitation [28]. NS is also crucial for preventing myoglobin-induced renal injury in EHS patients, a group prone to rhabdomyolysis [7]. This practice is widely recommended and used in clinical settings. In our study, NS was the most common intravenous fluid used across different treatment facilities. Patients in our study received an average of 3.02 ± 1.45 L of NS in the ED, a significantly larger volume than the 1.60 ± 1.10 L reported in Self et al.’s study [9].

However, the high chloride content in NS raises concerns. NS infusion appears to increase the need for intensive care, renal failure, prolonged hospital stays, and higher mortality rates [30–34]. Compared to balanced crystalloid, NS is associated with more frequent hyperchloremic metabolic acidosis and more frequent receipt and higher overall doses of vasopressors [30]. Critically ill patients receiving saline before ICU admission show a higher incidence of death and the need for new renal replacement therapies [31]. Limiting chloride-rich infusions in the ICU can reduce AKI and CRRT usage [16]. In our study, we found that the volume of NS infusion in the ED was associated with the incidence of AKI, ICU admission, and the length of ICU stay and hospitalization, even after adjusting for baseline serum creatinine level.

NS resuscitation may lead to hyperchloremic metabolic acidosis, contributing to renal vasoconstriction and reduced renal function [33–35]. Moreover, resuscitation with NS fails to repair endothelial glycocalyx thickness, inhibits syndecan-1 shedding, decreases tissue perfusion, and increases leukocyte rolling and adhesion [34], which aggravate tissue and organ damage. We observed that chloride levels rose with increased NS volumes, which is consistent with the previous studies [9, 15]. Furthermore, the chloride level after NS infusion, not the baseline chloride level, was significantly associated with the incidence of AKI. This suggests managing chloride levels is crucial in HS treatment because hyperchloremia is closely associated with metabolic acidosis and may lead to renal vasoconstriction and decreased glomerular filtration rate. Regarding AKI, in patients with HS, it is more reasonable to control the serum chloride level under 100 mmol/L. Numerous studies suggest that balanced crystalloids outperform NS, but their effectiveness specifically for HS patients remains uncertain. This area merits further investigation to determine the most effective treatment for HS.

This study is the first to investigate the relationship between NS infusion volume in the ED and AKI incidence in HS patients. We also examined the correlation between post-infusion blood chloride levels and AKI, revealing a near S-shaped curve. Given that completely avoiding NS infusion is challenging, managing blood chloride levels may be a more practical approach. Considering NS’s prevalence in EDs, healthcare providers should be aware of its safety implications. Effective HS treatment should balance immediate improvements with potential long-term effects. However, our study has limitations. Its retrospective design allows only for association analysis, not causality. We did not account for NS volumes administered post-ICU or general ward admission, nor did we consider chloride from other solutions, which could influence outcomes. Despite several sensitivity analyses, including genetic matching and random forest imputation, unobserved confounders might exist. We also lacked data on long-term outcomes post-discharge, important for understanding AKI’s progression and implications for quality of life and survival. Lastly, without classification by Acute Kidney Disease Network criteria [35], the study could not precisely delineate the relationship between NS volume and kidney damage.

## Conclusions

We conducted a multicenter retrospective cohort study to evaluate the association between the volume of NS infusion and AKI in adults with HS. We found that the volume of NS infusion was associated with a significant increase in the incidence of AKI, ICU admission, and length of stay in the ICU and hospital. Whether it would be beneficial to change chloride-rich intravenous fluids to chloride-poor fluids or to adopt a chloride-restrictive intravenous fluid strategy for HS patients needs further study.

## Data Availability

The datasets generated and/or analyzed during the current study are available from the corresponding author on reasonable request.
